# Conceptualizing Pathways of Sustainable Development in the Union for the Mediterranean Countries with an Empirical Intersection of Energy Consumption and Economic Growth

**DOI:** 10.3390/ijerph17155614

**Published:** 2020-08-04

**Authors:** Rashid Latief, Yusheng Kong, Yuanyuan Peng, Sohail Ahmad Javeed

**Affiliations:** 1 School of Finance and Economics, Jiangsu University, Zhenjiang 212013, China; 2 Department of Agricultural Economics, Purdue University, West Lafayette, IN 47909, USA; peng213@purdue.edu; 3 College of Finance, Nanjing Agricultural University, Nanjing 210095, China; 2017218009@njau.edu.cn

**Keywords:** energy consumption (EC), sustainable development (SD), economic growth (EG)

## Abstract

The availability of sufficient and trustworthy energy services at the reasonable cost in a securely and environmentally friendly manner, and conventionality with economic and social development requirements, is an important factor of sustainable development (SD). Energy plays a significant role in eliminating poverty and increasing living standards. However, most of the present energy forms of energy supply and consumption are unsustainable. This paper analyzes the association between economic growth (EG), energy consumption (EC), and sustainable development (SD) among other economic factors. The sample of 14 developed and developing member states of the Union for the Mediterranean (UFM) was selected. To deal with the endogeneity issue, the system- generalized method of moment (GMM) model was employed. Moreover, panel co-integration, Granger causality tests, and robustness tests were employed to examine the long-run and short-run causality among variables of interest. The results confirmed the short-run dynamic association from sustainable development (SD) to energy consumption (EC), and economic growth (EG) to sustainable development (SD). Moreover, the results validated the presence of long-run equilibrium association in the equations of EC and sustainable development (SD). The findings of this study will be supportive for the policymakers to formulate sustainable energy policies to stimulate the economic growth (EG) in the way of sustainable development (SD) in the UFM countries.

## 1. Introduction

This study aimed at investigating the dynamic association between economic growth (EG), energy consumption (EC), and sustainable development (SD) among other economic factors. We selected a sample of 14 developed and developing member states of the Union for the Mediterranean (UFM) and used data for the period 1995 to 2014.

The main motivation of this study is the lack of definite policies to achieve sustainable development in the countries. Earlier growth models of developed countries are based on exhaustive use of natural resources, but these growth models and policies are being questioned today. The major flaw of those models is to ignore the effect of environmental issues on the development of countries. To attain sustainable development, it is essential to adopt a balanced approach with economic, social, and environmental aspects [1]. On the other side, developing countries are also in a similar way of development as adopted by developed countries, but the environment of these countries could be severally affected. In a sustainable development strategy, the energy consumption (EC) has great importance, therefore, many countries are adopting policies to save energy, and these policies are based on the association between EC and EG [2].

The Mediterranean countries have energy benefits due to the extreme diversity in their energy resources, while the share of energy production of these countries is 11.4% as compared to the world energy production capacity. These countries cooperate related to energy production and distribution through the Maghreb Electricity Committee (Comelec). The other characteristics of Mediterranean countries are the disparity of energy consumption in the north-side and south-side. Energy consumption (EC) was reported double in the north side as compared to the south side of the Mediterranean region in 2009 [2].

The Mediterranean region has great potential for utilizing wind and solar energy sources to fulfill the energy demand. The energy mix in this region is dominated by fossil fuel, while renewable energy sources are not exploited well. Mediterranean countries have recently taken actions for implementing the strategies such as Mediterranean Solar Plant (MSP) and the Mediterranean Strategy for Sustainable Development (MSSD) to cope with energy and environmental challenges. The development of renewable energy projects in this region can give a lot of benefits, such as fulfilling the energy demand at a lower cost, attaining the sustainable economic growth, generating new employment opportunities, increasing the environmental quality, and increasing the cooperation between Mediterranean countries and European Union (EU) [3].

Because of the diversity of energy resources and its consumption level, social and environmental aspects, there is a need to formulate common and comprehensive policies on different energy issues and appropriate infrastructure. The energy issue is the major challenge for the Mediterranean countries to attain sustainable development. In 2008, the Union for the Mediterranean (UFM) countries was formed to increase the cooperation between the countries of the Mediterranean region and the EU. The purpose of this cooperation was to deal with energy and environmental challenges [2].

Previous studies have analyzed the connection between EC and EG both in developed and developing countries and found inconclusive findings. For example, Oh and Lee [4], Bowden and Payne [5], Karanfil [6] and Lise and Van Montfort [7] found unidirectional associations either from EG to EC or EC to EG. On the contrary, Belloumi [8] and Erdal, et al. [9] demonstrated a bidirectional association between EG and EC. This study focused on the UFM countries with the fact that these countries were given little attention in the literature. The literature on the causality between energy consumption (EC), economic growth (EG), sustainable development (SD), and other variables of UFM countries is quite limited as compared to other countries. However, to the best of our knowledge, no previous study has empirically examined the nexus of EC, EG, and sustainable development (SD), and this is the first systematic quantitative study that dealt with it.

To complete this study, we used different analysis techniques. Initially, we applied system GMM to overcome the endogeneity issue. Moreover, we tested the stationary of each variable by employing the unit root tests. After finding co-integration in models by using a panel co-integration test, we employed vector error correction model (VECM) and vector autoregression (VAR) to examine the causation between EC, EG, and sustainable development (SD) among other economic factors both in the long-run and short-run. To find more robust results, we employed impulse response analysis and variance decomposition analysis.

The following outcomes of this study are highlighted: EG and EC have shown bidirectional causality, while the sustainable development (SD) and EG have also shown bidirectional causality. Moreover, results have shown that sustainable development (SD) induces to EC in the short-run, while EG induces to sustainable development (SD) in the short-run. Furthermore, results demonstrated the existence of long-run equilibrium in the equations of EC and sustainable development (SD). These outcomes contribute to the literature by expanding the significance of EC and EG in the way of sustainable development (SD) in the UFM countries. This study could be helpful for these countries to reshape the policies for energy consumption, and other economic policies to find ways to increase economic growth and attain sustainable development (SD) in these countries.

This paper is organized as follows: the Section 2 reviews the previous literature. The Section 3 describes the synopsis of UFM countries. The Section 4 provides details of data collection, sample selection, and econometric techniques used. The Section 5 discusses the results of the study. The last section concludes the study.

## 2. Literature Review

Energy resources are generally considered compulsory for the development of society, while the sustainable development (SD) of society needs the supply of energy resources that are easily available for the long term at a reasonable cost and without harmful effects on society [10]. Energy helps in eradicating poverty and increasing the human welfare and living standards of people in society. However, the present forms of energy usage and its supply are considered unsustainable [11].

Many regions of the world do not have sustainable and secure energy resources which bound the economic expansion, while in the other regions, environmental pollution from the energy consumption constrains sustainable development [12]. Some energy resources such as fossil fuels and uranium are considered limited, while energy resources such as water, wind, and sunlight are sustainable for a long time. Moreover, wastes and biomass fuels are also considered as sources of sustainable energy resources [10,13].

Environmental concerns are the main issues that have to deal with countries in achieving sustainable development. Generally, the effect of environmental degrading activities is not sustainable for a long time, for instance, the cumulative effect of such activities on the environment creates problems related to health, ecological, and others. The major part of the environmental effects is connected with the consumption of energy resources. Preferably, societies want sustainable development with the consumption of energy resources that do not generate harmful environmental effects, but, in reality, all energy resources generate some harmful environmental effects. However, the harmful environmental effects can be overcome by increasing energy efficiency, because energy efficiency is strongly associated with environmental effects [14].

Many social concerns are also related to energy consumption, which includes demographic transition, education, poverty, indoor pollution, quality of life, and gender and age-related implications. The social aspect of sustainable development related to energy is the availability of basic energy services in the shape of commercial energy to people all over the world at an affordable cost. Energy indicators of social aspects have more significance for the developing countries that still have substantial portions of the population deprived of modern energy services [12].

The accessible and secure energy resources are essential for fortifying the economic growth. All sectors of the economy such as agricultural, residential, service, and others depend upon sustainable energy resources. Many economic activities including industrial growth, job opportunities, rural and urban development are strongly influenced by the energy contribution. The availability of electricity is most important in many production activities, distribution of information, and other industries. The energy indicators in the economic aspect reflect two themes: the ways of usage and production, and secure supply. The first theme of usage and production comprises of the sub-issues such as supply efficiency, usage, production, energy mix, and prices. The second theme of secure supply consists of reliance on supply and energy stocks [12].

The discussion about the association between energy and EG is continued among economists for a long time, but they could not find conclusive evidence about it yet. On one side, neoclassical economists believe that energy is not an important factor that originates the EG, they argue that energy affects EG only in certain ways [15,16]. On the other side, ecological economists consider energy as an imperative factor of production in line with the Laws of Thermodynamics and proposed a model for it [17]. Afterward, many other researchers also endorsed their findings of the association between economic production and energy [18,19].

The debate about the association between EC and EG revolves around four hypotheses: (1) Growth hypothesis, (2) Conservation hypothesis, (3) Neutral hypothesis, and (4) Feedback hypothesis.

**Growth Hypothesis**: this hypothesis refers to the unidirectional causality between EC and EG, causality running from EC to EG. It infers that a decrease of EC may lead to a decline in EG, while an increase of EC can promote EG [20]. Besides the labor and capital, energy is an essential factor of output, while insufficient energy supply and energy supply shocks can restrict economic growth. From the empirical point of view, Soytas and Sari [21] found that Turkey has a one-way Granger causality from power consumption to manufacturing growth. Gurgul and Lach [22] used quarterly data from 2000 to 2009 to analyze the association between total EC and EG in Poland and found one-way causality from EC to EG in Poland. Chang, et al. [23] found one-way causation from EC to output in Taiwan Province of China by using the VECM model.
**Conservation Hypothesis:** The proponents of the conservation hypothesis argue in the favor of unidirectional causation from EG to EC [24]. If EG causes EC, it shows that EG of the country does not depend on energy, so energy conservation policies will not negatively affect EG. From the empirical perspective, Ghosh [25] used the annual data of India from 1950 to 1997 and found a causal connection between EG and EC (electricity), but the reverse relationship does not exist. Ghali and El-Sakka [26] used data from Canada and established one-way causation between output growth and EC. Ang [27] used the data of Malaysia from 1971 to 1999 and confirmed one-way causation from EG to EC.
**Neutral Hypothesis:** This hypothesis asserts that there is negligible or no effect of EC on EG [28]. If EC and EG do not cause each other, it infers that energy conservation or energy conservation policies will not affect EG, and the acceleration or deceleration of EG will not have a relevant effect on EC. From the empirical point of view, Ferguson, et al. [29] could not find causation between EC and EG by using the data of seven countries. Altinay and Karagol [30] used Hsiao’s causality test and data of Turkey from 1950 to 2000 and found no causality between EC and EG. Fatai, et al. [31] used Toda and Yamamoto tests to examine the data of New Zealand from 1960 to 1999, and findings of their studies supported the independent relationship between EC and EG.
**Feedback Hypothesis:** This hypothesis postulates that EC and EG have bi-directional causality with each other [20]. If EC and EG bi-directionally cause each other, it infers that EC and EG are mutually affected, and any change in one aspect will cause corresponding changes in the other. From the empirical point of view, Glasure and Lee [32] found two-way causality between EC and EG through multiple VAR models. Yang [33] examined the association between EG and EC and found that there was a two-way causality between EC and EG by using data of India. Erdal, Erdal and Esengün [9] found two-way interactions between EC and EG by using data of Turkey from 1970 to 2006.

Theoretically, the association between EC and EG has been explored in the literature based on different theories. For instance, Xiang and Diqing [34]] examined the intrinsic relationship between natural resources, environmental pollution, and EG based on the endogenous growth theory. Xiaobo [35] studied different energy factors based on the theory of Copeland and Taylor [36]. Xepapadeas [37] explored the association between resources, the environment, and EG based on the Solow model, and pointed out that sustainable growth and environmental protection can be achieved simultaneously under certain conditions. Zuo and Ai [38] investigated the association among EC, environment, human capital, technological innovation, and EG based on dynamic optimization theory.

Empirically, researchers have used different empirical analysis techniques to examine the association between EC and EG. For instance, Lazzaretto and Toffolo [39] and Tashimo and Matsui [40], studied the 3E system composed of environment, EC and EG by using data envelopment analysis methods. Hawdon and Pearson [41], and Oliveira and Antunes [42] analyzed the interaction between environmental pollution, EC, and EG by using the input-output method. Zhao [43] studied the association between EC, EG, and environmental pollution by using system coordination and fuzzy mathematics. Cui and Wang [44] and Xia and Xu [45] examined the association between EC and EG by applying different measurement models such as VAR, co-integration, and VECM.

## 3. Synopsis of the Union for the Mediterranean (UFM) Countries

By European standards, Albania is a relatively poor and economically backward country. Albania is steadily transitioning to a more modern open-market economy. In 2018, Albania had a real growth rate of GDP about 3.5%, and the per capita GNP was US $13,274. The sector-wise GDP was distributed among the agriculture (21.6%), industry (14.9%) and service (63.5%) sectors. The main export industries include textiles, footwear, asphalt, metal, non-metal minerals, crude oil, vegetables, fruits, and tobacco, etc. At present, agriculture accounts for about one-fifth of the GDP, while service industries such as tourism account for more than half of the GDP. It is important to recognize that in 2003 and 2004, the domestic economy of Albania grew strongly, while the country had a lot of oil and gas resources, and there was no inflation problem in the country.

Bulgaria is an agricultural country, with roses, yogurt, and wine enjoying a great reputation in the international market. At present, food processing and textile industries are the main industries, while the tourism industry has developed in the past few years. Bulgaria is a member of the China-EU free trade agreement. The strongest sectors of the economy comprised of energy, mining, metallurgy, machinery manufacturing, agriculture, and tourism. The main industrial exporting products include clothing, steel, machinery, and refined fuel. The economy of Bulgaria has grown rapidly in recent years, and the per capita GDP of Bulgaria was US $20,116 in 2016 [46].

The economy of Croatia is dominated by the tertiary industry. Tourism, construction, shipbuilding, pharmaceutical, and other industries are highly developed in this country. This country has a lot of forest and water resources, with a national forest area of 2,232,000 hectares. Besides, Croatia has oil, natural gas, aluminum, and other resources. The main industries include chemical, plastic, mechanical parts, metal, electronic parts, crude steel, aluminum, paper, wood, building materials, textiles, shipbuilding, oil, tourism, food and beverage, and the main export industries comprise of vehicles, machinery, textiles, chemicals, food, and fuel.

The Czech Republic was listed as a developed country by the World Bank in 2006. By the end of 2019, the GDP of the Czech Republic was reported more than US $22,000. Machinery manufacturing, chemical industry, metallurgy, and other industrial sectors are highly developed in the Czech Republic. Foreign trade, tourism, and government financing are the main economic pillars of the country, and these are the main driving forces for the stable growth of the domestic economy. The important fact is that the Czech Republic is no longer a developing country but is steadily included in the list of the 30 most developed countries. The Czech Republic has plentiful resources of lignite, hard coal, and uranium, while it has also mineral resources such as manganese, aluminum, zinc, fluorite, graphite, and kaolin.

The Egyptian economy is among the highly diversified economies of the Middle East. Various important industries contribute to the economy almost equally. Egypt is also considered to be an influencing power of Islamic faith in the Mediterranean and the Middle East areas. The economy of Egypt is mainly dependent on agriculture, oil exports, tourism, and labor exports. Oil is a very important part of the Egyptian minerals. The origin of the oil-producing area is on the west coast of the Red Sea, but the production of this area has been gradually reduced. In 2018, the gross domestic product (PPP) of Egypt was the US $1105.039 billion, with an average per capita of US $13,759 [47].

Before 2006, Estonia had a strong economy with an annual growth rate of 10%. Estonia has been pursuing a free economic policy, vigorously implementing privatization, and free trade policy. Estonia is ranked at 1st in the EU Member States with rapid economic development and its annual economic growth rate. Estonia is almost energy independent country, with more than 90% of the electricity demand provided by locally mined oil shale. The main mineral resources consist of oil shale, peat, phosphate rock, limestone, etc. Estonia imports oil products from Western Europe and Russia.

Hungary has a high-income mixed economy of the OECD, with an output value of US $265.307 billion, measured by purchasing power parity. Hungary has an export-oriented economy and focused on international trade, therefore, Hungary is the 36th largest export-oriented country in the world. Hungary has a private economy of more than 80% with an overall tax rate of 39.1%. The main industries include pharmaceutical, motor vehicles, chemical, metallurgy, electrical appliances, and tourism.

In 2016, the GDP in purchasing power parity (PPP) of Lithuania was estimated at US $85.435 billion, with a per capita value of US $29,716 [48]. Agriculture is dominated by high-level animal husbandry, which accounts for more than 90% of the output value of agricultural products. The major crops produced by Lithuania include flax, potato, beet, and various vegetables. Lithuania is rich in amber, with a small amount of clay, sandstone, lime, gypsum, peat, iron ore, apatite, and oil, and it imports oil and natural gas from other countries. A small amount of oil and gas resources have been found in the western coastal areas of Lithuania, but the reserves have not yet been proven. The major industries of Lithuania comprise of mining and quarrying industry, processing and manufacturing industry and energy industry. Some industries are relatively developed, mainly including food, wood processing, textile, chemical industry, etc., while, machinery manufacturing, chemical, petrochemical, electronic, and metal processing industries are developing rapidly.

In 2018, the GDP (PPP) of Morocco was the US $315.441 billion with US $8959 per capita GDP [47]. Major economic sectors of Morocco consist of tourism, fisheries, and phosphate minerals. Morocco has plenty of phosphate reserves about 110 billion tons and ranked 1st in the world. The agriculture and animal husbandry industries are greatly affected by climate change. The economy of Morocco relies on external financing in many ways, while France and Spain are the largest donors to help the economy of Morocco. The mining industry of Morocco has a good development momentum, mainly due to the increasing demand for phosphate in the international market. Chemical, automobile, aviation, electronics, and other industries have become the major helping hand for the development of the manufacturing industry. However, due to the poor performance of the textile, and leather manufacturing industries, the overall growth of the manufacturing industry was slow.

According to the information released by the Central Statistical Office of Poland, the per capita GDP of Poland was US $13,414 with an annual growth rate of 4.6% in 2017. In 2019, the real economic growth rate of Poland was 4.1%, and the nominal GDP was US $589.847 billion. Poland is rich in mineral resources such as coal, shale gas, sulfur, copper, zinc, lead, aluminum, and silver. Poland is the largest producer of hard coal in central Europe, and its output can meet 10% of the EU’s total demand. Poland is ranked 9th in Europe in terms of lignite extraction, but only 15% of its reserves are developed. Poland has also specific hydrocarbon fuels. By the end of 2017, 9.513 million hectares of forest (green space) was covered, with a forest coverage rate of 30.4 percent. The main industrial products of Poland include coal, steel, cars, cement, and so on.

The economy of Romania is considered among the top economies of Central and Eastern Europe. The economy of Romania was rapidly growing, and its overall performance was excellent in 2015. Romania made a lot of gratifying progress, not only in terms of macroeconomic indicators but also in terms of microeconomic level. In 2014, many policies were transformed into specific actions in favor of entrepreneurs and businessmen, especially in terms of employment promotion. In 2017, the economic growth of Romania was reached 7%, even it exceeded the Chinese economic growth (6.9%) [47].

The Slovak Republic was an agricultural country, and there was no basic industry in the country in the early years. The Czechoslovak Communist Party gradually established steel, food processing, and military industries in Slovakia during its administration, and narrowed the economic gap with the Czech Republic. The economy of the Slovak Republic was declined in 2009 due to the international financial crisis, but it recovered growth in 2010 and beyond. The automobile industry is the industrial pillar of the Slovak national economy. The Slovak Republic is not a rich country in terms of oil and gas resources, and it mostly has small oil fields, which are scattered in the Carpathian Mountains and the eastern region.

Since the government of Ben Ali was overthrown in 2011, the Tunisian economy was severely affected. In 2014, the economic growth of Tunisia was only 1%. By 2015, the unemployment rate in Tunisia had risen to nearly 30%, which was twice the unemployment rate during the administration of Ben Ali. Until 2016, the Tunisian economy was recovered strongly. In 2016, the total GDP of Tunisia was the US $130.77 billion, with a per capita GDP of US $11,651 [48]. Tunisia is rich in olive oil, it is known as the “olive oil garden of the world” and “the country of olives”. Renewable energy plays a secondary role in the energy supply, while solar energy is widely used in Tunisia. Photovoltaic power generation, wind power generation, etc. bring great impetus to the development of the Tunisian national economy.

The economic status of Turkey is so important in the world, and it is a founding member of the Organization for Economic Cooperation and Development (OECD). According to the World Bank, the per capita GDP of Turkey was reached $766.5 billion in 2018, ranked at 19th in the world. At the same time, Turkey is rich in natural and mineral resources. Turkey has more than 60 kinds of mineral resources. According to the statistics of mineral diversity, Turkey is ranked 10th in the world in terms of minerals. Turkey has plentiful reserves of boron salt, account for 72% of the world. In addition to boron salt reserves, Turkey is also rich in coal, iron, copper, and chromium.

## 4. Material and Methods

### 4.1. Sample and Data Sources

**Figure 1:**
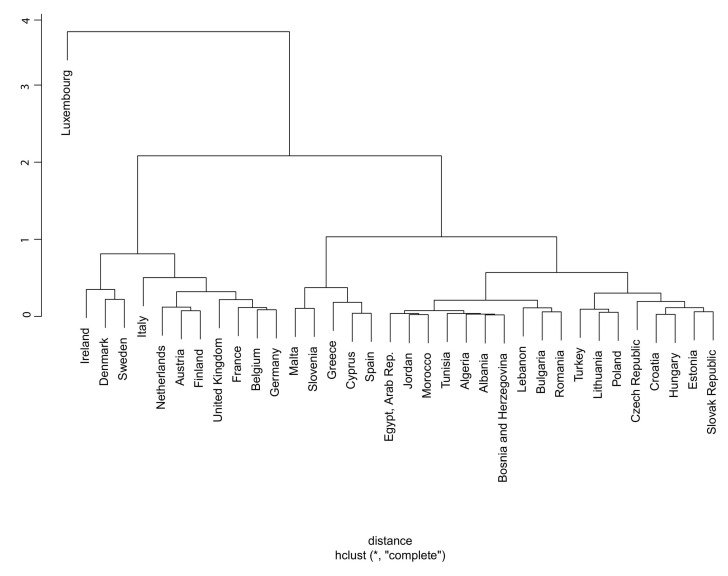
Hierarchical Cluster Analysis (HCA) dendrogram analysis.

We selected the sample of 14 developed and developing member states of the Union for the Mediterranean (UFM) (a list of all countries which are members of the Union for Mediterranean (UFM) countries is available here [49]), whose GDP per capita does not exceed $25,000 during the time duration from 1995 to 2014, by following the sampling method adopted by Comes, et al. [50]. The countries include Albania, Bulgaria, Croatia, Czech Republic, Egypt, Estonia, Hungary, Lithuania, Morocco, Poland, Romania, Slovak Republic, Tunisia, and Turkey. We used these countries based on Hierarchical Cluster Analysis (HCA), shown in Figure 1. The figure is drawn by using Ward-hierarchical grouping method [51] and hclust-clustering package [52] from R (R Core Team, Vienna, Austria) [53]. We collected annual data on EG, EC, sustainable development, FDI, international trade, labor force, capital stock, financial development, population and inflation from the World Bank database and International Monetary Fund (IMF), International Financial Statistics (IFS) for the period 1995 to 2014.

### 4.2. Empirical Models

To find the association among EC, EG and sustainable development (SD) in the UFM countries, in line with prior studies [2,54,55], this study considers the following equations for estimations:

(1) $$LnEG_{i,t}=\alpha_{i}+\beta_{1}LnEC_{i,t}+\beta_{2}LnSD_{i,t}+\beta_{3}LnCF_{i,t}+\beta_{4}LnLF_{i,t}+\beta_{5}LnFDI_{i,t}+\beta_{6}LnINF_{i,t}+\mu_{i,t}$$

(2) $$LnEC_{i,t}=\alpha_{0}+\beta_{1}LnEG_{i,t}+\beta_{2}LnSD_{i,t}+\beta_{3}LnCF_{i,t}+\beta_{4}LnLF_{i,t}+\beta_{5}LnPOP_{i,t}+\beta_{6}LnFD_{i,t}+\lambda_{i,t}$$

(3) $$LnSD_{i,t}=\varepsilon_{0}+\beta_{1}LnEC_{i,t}+\beta_{2}LnEG_{i,t}+\beta_{3}LnCF_{i,t}+\beta_{4}LnLF_{i,t}+\beta_{5}LnFDI_{i,t}+\beta_{6}LnTR_{i,t}+\nu_{i,t}$$

From Equations (1) to (3), $\alpha_{i}$, $\alpha_{0}$ and $\varepsilon_{0}$ are the intercept terms; $LnEG_{i,t}$ is the logarithm of economic growth; $LnEC_{i,t}$ is the logarithm of energy consumption; $LnSD_{i,t}$ is the logarithm of sustainable development; $LnCF_{i,t}$ is the capital formation; $LnLF_{i,t}$ is the logarithm of labor force; $LnFDI_{i,t}$ is the logarithm of foreign direct investment; $LnPOP_{i,t}$ is the logarithm of total population of the country; $LnFD_{i,t}$ is the logarithm of financial development; $LnTR_{i,t}$ is the logarithm of international trade; $LnINF_{i,t}$ is the logarithm of inflation; $\mu_{i,t}$, $\lambda_{i,t}$, and $\nu_{i,t}$ are error terms. Subscript *i* and *t* denote countries and periods respectively. The three way association among economic variables is broadly analyzed by Equations (1)–(3). The Equation (1) posits that sustainable development (SD), capital formation (CF), labor force (LF), FDI, and international trade (TR) are the potential determining factors of EG as suggested by Esseghir and Khouni [2] and Boţa-Avram, et al. [56]. Equation (2) postulates that economic growth (EG), sustainable development (SD), CF, LF, POP and financial development (FD) are the potential determining factors of energy consumption (EC) as recommended by [Anwar and Sun [57], and Lee [58]]. Equation (3) posits that EC, EG, capital formation (CF), labor force, FDI and international trade (TR) are the potential determining factors of sustainable development (SD) as recommended by [Boţa-Avram, Groşanu, Răchişan and Gavriletea [56]]. We assume that all variables are stationary, the Equation (1) entails the panel co-integration with both panel-VECM and panel-VAR as follows:

For VECM:

(4) $$∆lnEG_{it}=\varphi_{1}ecm_{i,t-1}+\delta_{11}∆lnEC_{it}+\delta_{21}∆lnSD_{it}+\delta_{31}∆lnCF_{it}+\delta_{41}∆lnLF_{it}+\delta_{51}∆lnFDI_{it}+\delta_{61}∆lnINF_{it}+\in_{1it}$$

(5) $$∆lnEC_{it}=\varphi_{2}ecm_{i,t-1}+\delta_{12}∆lnEG_{it}+\delta_{22}∆lnSD_{it}+\delta_{32}∆lnCF_{it}+\delta_{42}∆lnLF_{it}+\delta_{52}∆lnFDI_{it}+\delta_{62}∆lnINF_{it}+\in_{2it}$$

(6) $$∆lnSD_{it}=\varphi_{3}ecm_{i,t-1}+\delta_{13}∆lnEG_{it}+\delta_{23}∆lnEC_{it}+\delta_{33}∆lnCF_{it}+\delta_{43}∆lnLF_{it}+\delta_{53}∆lnFDI_{it}+\delta_{63}∆lnINF_{it}+\in_{3it}$$

(7) $$∆lnCF_{it}=\varphi_{4}ecm_{i,t-1}+\delta_{14}∆lnEG_{it}+\delta_{24}∆lnEC_{it}+\delta_{34}∆lnSD_{it}+\delta_{44}∆lnLF_{it}+\delta_{54}∆lnFDI_{it}+\delta_{64}∆lnINF_{it}+\in_{4it}$$

(8) $$∆lnLF_{it}=\varphi_{5}ecm_{i,t-1}+\delta_{15}∆lnEG_{it}+\delta_{25}∆lnEC_{it}+\delta_{35}∆lnSD_{it}+\delta_{45}∆lnCF_{it}+\delta_{55}∆lnFDI_{it}+\delta_{65}∆lnINF_{it}+\in_{5it}$$

(9) $$∆lnFDI_{it}=\varphi_{6}ecm_{i,t-1}+\delta_{16}∆lnEG_{it}+\delta_{26}∆lnEC_{it}+\delta_{36}∆lnSD_{it}+\delta_{46}∆lnCF_{it}+\delta_{56}∆lnLF_{it}+\delta_{66}∆lnINF_{it}+\in_{6it}$$

(10) $$∆lnINF_{it}=\varphi_{7}ecm_{i,t-1}+\delta_{17}∆lnEG_{it}+\delta_{27}∆lnEC_{it}+\delta_{37}∆lnSD_{it}+\delta_{47}∆lnCF_{it}+\delta_{57}∆lnLF_{it}+\delta_{67}∆lnFDI_{it}+\in_{7it}$$

For the VAR Model:

(11) $$lnEG_{it}=\eta_{0}+\eta_{1}lnEG_{i,t-k}+\eta_{2}lnEC_{i,t-k}+\eta_{3}lnSD_{i,t-k}+\eta_{4}lnCF_{i,t-k}+\eta_{5}lnLF_{i,t-k}+\eta_{6}lnFDI_{i,t-k}+\eta_{7}lnINF_{i,t-k}+\psi_{1it}$$

(12) $$lnEC_{it}=\theta_{0}+\theta_{1}lnEC_{i,t-k}+\theta_{2}lnEG_{i,t-k}+\theta_{3}lnSD_{i,t-k}+\theta_{4}lnCF_{i,t-k}+\theta_{5}lnLF_{i,t-k}+\theta_{6}lnFDI_{i,t-k}+\theta_{7}lnINF_{i,t-k}+\psi_{2it}$$

(13) $$lnSD_{it}=\kappa_{0}+\kappa_{1}lnSD_{i,t-k}+\kappa_{2}lnEG_{i,t-k}+\kappa_{3}lnEC_{i,t-k}+\kappa_{4}lnCF_{i,t-k}+\kappa_{5}lnLF_{i,t-k}+\kappa_{6}lnFDI_{i,t-k}+\kappa_{7}lnINF_{i,t-k}+\psi_{3it}$$

(14) $$lnCF_{it}=\upsilon_{0}+\upsilon_{1}lnCF_{i,t-k}+\upsilon_{2}lnEG_{i,t-k}+\upsilon_{3}lnEC_{i,t-k}+\upsilon_{4}lnSD_{i,t-k}+\upsilon_{5}lnLF_{i,t-k}+\upsilon_{6}lnFDI_{i,t-k}+\upsilon_{7}lnINF_{i,t-k}+\psi_{4it}$$

(15) $$lnLF_{it}=\rho_{0}+\rho_{1}lnLF_{i,t-k}+\rho_{2}lnEG_{it-k}+\rho_{3}lnEC_{i,t-k}+\rho_{4}lnSD_{i,t-k}+\rho_{5}lnCF_{i,t-k}+\rho_{6}lnFDI_{i,t-k}+\rho_{7}lnINF_{i,t-k}+\psi_{5it}$$

(16) $$lnFDI_{it}=\omega_{0}+\omega_{1}lnFDI_{i,t-k}+\omega_{2}lnEG_{i,t-k}+\omega_{3}lnEC_{i,t-k}+\omega_{4}lnSD_{i,t-k}+\omega_{5}lnCF_{i,t-k}+\omega_{6}lnLF_{i,t-k}+\omega_{7}lnINF_{i,t-k}+\psi_{6it}$$

(17) $$lnINF_{it}=\vartheta_{0}+\vartheta_{1}lnINF_{i,t-k}+\vartheta_{2}lnEG_{i,t-k}+\vartheta_{3}lnEC_{i,t-k}+\vartheta_{4}lnSD_{i,t-k}+\vartheta_{5}lnCF_{i,t-k}+\vartheta_{6}lnLF_{i,t-k}+\vartheta_{7}lnFDI_{i,t-k}+\psi_{7it}$$

From the Equations (4) to (10), $ecm_{i,t-1}$ are the error correction term; $\varphi_{1}$, $\varphi_{2}$, $\varphi_{3}$, $\varphi_{4}$, $\varphi_{5}$, and $\varphi_{6}$ are for capturing the long-run connection among variables. ∆s represents the difference operators. $\varphi_{1}<0$, $\varphi_{2}<0$, $\varphi_{3}<0$, $\varphi_{4}<0$, $\varphi_{5}<0$, and $\varphi_{6}<0$ assume that long run connection does not obstruct fluctuations in EG, EC, sustainable development (SD), capital formation (CF), labor force, FDI, and inflation, whereas the greater sign exhibits opposite meaning of it. $\in_{1t}$, $\in_{2t}$, $\in_{3t}$, and $\in_{4t}$ denote the error terms. From the Equations (11) to (17), $\eta_{0}$, $\theta_{0}$, $\kappa_{0}$, $\upsilon_{0}$, $\rho_{0}$, $\omega_{0}$, *ϑ*_0_ are the intercept terms; k is the number of lags; $\psi_{1it}$, $\psi_{2it}$, $\psi_{3it}$, $\psi_{4it}$, $\psi_{5it}$, and $\psi_{6it}$ denote the error terms.

### 4.3. Estimation Techniques

Generally, the problem of endogeneity occurs in the panel dataset as a result of the mutual correlation between endogenous variables and stochastic error terms. Consequently, we used a Durbin–Wu–Hausman test to check the endogeneity bias in our dataset. To deal with endogeneity bias, we employed system-GMM instrumental variables estimation technique. Moreover, we tested the stationary of each variable by employing the unit root test. After finding co-integration in the models by using a panel co-integration test, we employed VECM and VAR models to find the causation among EC, EG, and sustainable development among other economic factors. To find more robust results, we employed impulse response analysis (IRA) and variance decomposition analysis (VDA). All variables are described with measures and data sources in Table 1.

## 5. Results and Discussion

### 5.1. Summary Statistics

Table 2 describes the summary statistics of EG, EC, sustainable development (SD), and other control variables used in this study.

### 5.2. Unit Root Test

Table 3 exhibits the results of panel unit root tests such as Levin-Lin (LL), Im-Pearson-Shin (IPS), Fisher Augmented-Dickey Fuller (ADF) test, and Fisher Philips-Perron (PP). The results of all unit root tests indicate that all variables including EG, EC, sustainable development (SD), and others are found to be non-stationary at level and converted into stationery with the help of first difference of these variable. These results encourage us to employ a panel co-integration test to find the association between these variables.

### 5.3. System-Generalized Method of Moments (GMM)

Table 4 displays the results of the system GMM to investigate the dynamic association between EG, EC, and sustainable development (SD) among other factors. The results of Model 1 indicate that EC has a significant positive association with EG at 1% level. The economic significance implies that a rise in EC by 1% can increase the EG by 0.3111%. Moreover, SD has shown a significant negative association with EG at 1% level. The estimated coefficient of this result specifies that a 1% rise in the SD can decrease the EG by 0.0822%. From the controlling variables, CF has shown a significant positive association with EG, while LF has a significant negative association with EG.

The results of Model 2 demonstrate that EG has a significant positive effect on EC at 1% level. The estimated coefficient specifies that a 1% rise in EG can increase EC by 0.9640%. From the controlling variables, CF and POP have demonstrated a significant negative association with EC, while LF and FD have shown a significant positive association with EC. The results of Model 3 postulate that EG has a significant negative association with SD at 1% level. This result demonstrates that 1% rise in EG decreases the SD by 0.9633%. From the controlling variables, CF has shown a significant positive association with SD, while LF has shown a significant negative association with SD.

### 5.4. Co-integration Tests

Before using VECM, we employed a panel cointegration test with different methods including Pedroni and Kao to find the cointegration between EG, EC, sustainable development (SD), and other factors. The results of the panel cointegration test suggest that all these variables are cointegrated with each other in all models (1–3), results are shown in Table 5. This finding encourages us to employ VECM to find the causality among variables.

### 5.5. Vector Error Correction Model (VECM)

Table 6 demonstrates the results of VECM used to inspect the direction of causality between variables used in the model (1) of EC, EG, and sustainable development (SD). The results of Model (4), the coefficient of error correction term (ECT) is exposed to be insignificant, while the labor force induces short-run dynamic association with EG at 5% level. It suggests that the growth in the labor force can stimulate the EG. The results of Model (5), the coefficient of ECT is exposed to be significant at 1% level with a negative sign (i.e., ECT is −0.0238). It suggests that the speed of adjustment (SOA) of energy consumption (EC) is 2.38% towards long-run equilibrium. Additionally, the short-run dynamic association is revealed from sustainable development (SD) to EC at 1% level.

The results of Model (6), the coefficient of ECT is exposed to be significant at 1% level with a negative sign (i.e., ECT is −0.1489). It postulates that SOA of sustainable development (SD) is 14.89% towards the long-run equilibrium. Besides, the dynamic association is revealed in the short run from economic growth (EG) and capital formation (CF) to sustainable development (SD) at 5% and 10% levels respectively. The results of Model (7), the coefficient of ECT is exposed to be insignificant, while the energy consumption (EC) induces the short-run dynamic association with capital formation (CF) at 1% level.

The results of Model (8), the coefficient of ECT is exposed to be significant at 10% level with a negative sign (i.e., ECT is −0.0104). It suggests that the SOA of the labor force (LF) is 1.04% towards long-run equilibrium. Additionally, the short-run dynamic association is revealed from EG, EC, sustainable development (SD), and inflation (INF) to the labor force (LF) at 5%, 1%, 10%, and 5% levels respectively. The results of Model (9), the coefficient of ECT is exposed to be significant at 5% level with a positive sign (i.e., ECT is 0.3549). It postulates that SOA of FDI is 35.49% towards long-run equilibrium. Moreover, EC induces the short-run dynamic association with FDI at 1% level. The results of Model (10), the coefficient of ECT is exposed to be significant at 10% level with a negative sign (i.e., ECT is 0.8983). It suggests that SOA of inflation (INF) is 89.83% towards long-run equilibrium.

### 5.6. Vector Autoregression (VAR) Model

Table 7 shows the results of Models (11) to (17) estimated by employing a panel VAR model. The results of Model (11) indicate that economic growth (EG) lagged by 1 period significantly positively influences EG at 1% level. It suggests that an increase in the EG lagged by 1 period can stimulate the EG by 92.18%. Moreover, energy consumption (EC) lagged by 1 period significantly positively influences EG at 5% level. It implies that an additional input in the EC lagged by 1 period can increase the EG by 5.27%.

The results of Model (12) specify that EC lagged by 1 period significantly positively influences the EC at 1% level. It postulates that an increase in the EC lagged 1 period can increase the EC by 98.59%. The results of Model (13) show that EC lagged by 1 period significantly negatively influences sustainable development (SD) at 1% level. It suggests that an additional input in the EC lagged by 1 period can decrease the SD by 17.71%. Furthermore, SD lagged by 1 period significantly positively influences the SD at 1% level. It infers that a rise in the SD lagged by 1 period can increase the SD by 65.71%. Additionally, inflation (INF) lagged by 1 period significantly negatively influences the SD at 10% level. It postulates that the growth in inflation (INF) lagged by 1 period can reduce the SD by 4.51%.

The results of Model (14) imply that SD lagged by 1 period significantly positively influences capital formation (CF) at 10% level. It implies that an increase in the SD lagged by 1 period can stimulate the CF by 3.55%. Moreover, CF lagged by 1 period significantly positively influences the CF at 1% level. It suggests that an additional input in CF lagged by 1 period can increase capital formation by 86.18%. Furthermore, the labor force (LF) lagged by 1 period significantly positively influences CF at 5% level. It implies that growth in LF lagged by 1 period can stimulate CF by 14.35%. Additionally, FDI lagged by 1 period significantly positively influences CF at 5% level. It suggests that a rise in FDI lagged by 1 period can intensify CF by 3.33%.

The results of Model (15) indicate that EG lagged by 1 period significantly negatively influences the LF at 5% level. It suggests that growth in EG lagged by 1 period can reduce the LF by 1.48%. Furthermore, EC lagged by 1 period significantly negatively influences the LF at 5% level. It implies that an additional input in the EC can reduce the LF by 0.75%. Moreover, CF lagged by 1 period significantly positively influences the LF at 1% level. It postulates that growth in CF lagged by 1 period can increase the LF by 1.65%. Additionally, LF lagged by 1 period significantly positively influences the LF at 1% level. It suggests that growth in LF lagged by 1 period can increase the labor force by 98.83%. Moreover, FDI lagged by 1 period significantly influences the LF at 10% level. It implies that a rise in FDI lagged by 1 period can increase the LF by 0.22%.

The results of Model (16) specify that EC lagged by 1 period significantly positively influences FDI at 1% level. It postulates that growth in EC lagged by 1 period can raise the level of FDI by 51.44%. Moreover, FDI lagged by 1 period significantly positively influences FDI at 1% level. It implies that a rise in FDI lagged by 1 period can increase the level of FDI by 60.92%. Furthermore, inflation (INF) lagged by 1 period significantly negatively influences the FDI at 5% level. It suggests that an increase in the INF lagged by 1 period can decrease the level of FDI by 11.93%.

The results of Model (17) indicate that EG lagged by 1 period significantly negatively influences inflation (INF) at 5% level. It suggests that a rise in the EG lagged by 1 period can reduce the INF by 40.53%. Moreover, sustainable development (SD) lagged by 1 period significantly negatively influences INF at 5% level. It postulates that an additional input in the SD lagged by 1 period can reduce the INF by 14.32%. Furthermore, INF lagged by 1 period significantly positively influences INF at 1% level. It implies that an increase in the INF lagged by 1 period can escalate the INF by 68.13%.

### 5.7. Variance Decomposition Analysis (VDA)

Table 8 demonstrates the results estimated by VDA for the Model (1) related to economic growth (EG). Results show that EG has a short-run self-explanatory effect, however, the long-run effect is decreased to 89.15%. Energy consumption (EC), sustainable development (SD), CF, LF, FDI, and INF did not highlight shocks in the short run. Besides, 1.3% of EC is expounded by shocks to EG in the long run. Furthermore, EG is influenced by 1.29% shocks of SD in the long run. Likewise, 0.23% of CF is explicated by shocks to EG in the long run. Additionally, 0.10% of LF is expounded by shocks to EG in the long run. Besides, EG is influenced by 2.40% shocks of FDI in the long run. Moreover, 5.53% of inflation (INF) is explicated by shocks to EG in the long run.

### 5.8. Impulse Response Analysis (IRA)

**Figure 2:**
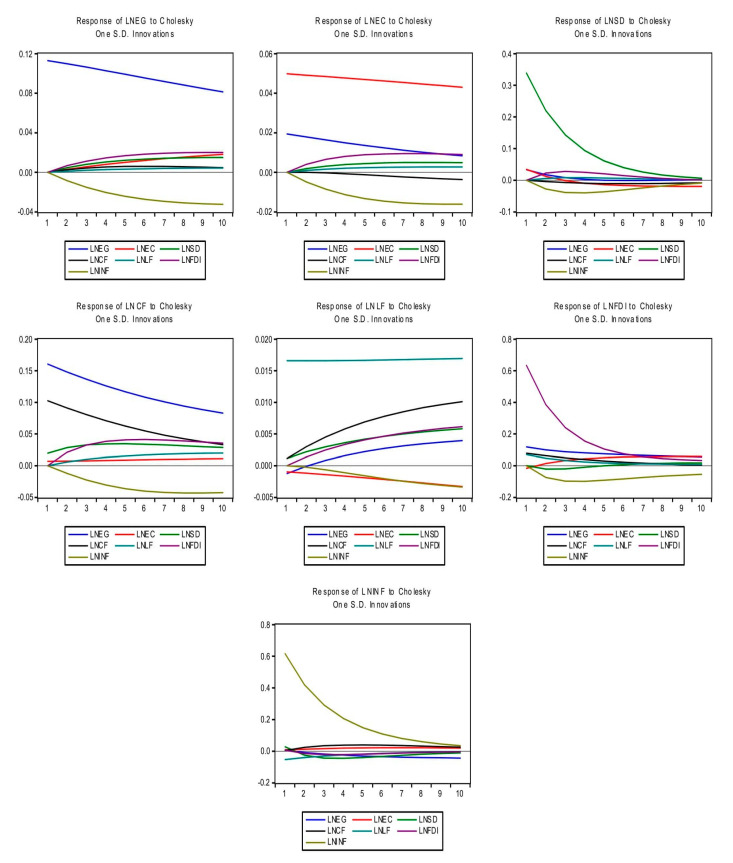
Impulse Response Analysis (IRA).

Figure 2 depicts the estimated shocks of variables by IRA which highlights the influence of one variable on the one unit of impulse in other variables. IRA is employed by keeping in view the order of variables with ten years period. Figure 2 shows that shocks in energy consumption (EC), sustainable development (SD), capital formation (CF), the labor force (LF) and FDI demonstrated positive connection with the EG over the period, whereas the shocks in the inflation (INF) exhibited the negative connection with EG over the period.

Figure 2 illustrates that the shocks in EG, SD, LF, and FDI highlighted positive connection with EC over the period, whereas the shocks in INF and CF demonstrated a negative connection with EC over the period. Moreover, the shocks in the EG, LF, and FDI showed a positive connection with SD over the period, whereas the shocks in CF and INF exposed negative connection with SD over the period. While, the shocks in EC demonstrated a positive effect on SD in four years at the starting period, while these shocks showed a negative effect on SD in the rest of the period.

Figure 2 shows the shocks in all concerned variables except inflation (INF) exhibited a positive influence on CF over the period. Furthermore, shocks in EG, SD, CF, and FDI exposed positive effects on LF over the period, whereas shocks in EC and INF demonstrated a negative influence on LF over the period. Moreover, shocks in EG, EC, CF, and LF showed a positive association with FDI, whereas shocks in SD demonstrated a negative association with FDI at the starting years (1–5), but later these shocks showed a positive association with FDI for the rest of period. While the shocks in INF demonstrated a negative connection with FDI over the period. Furthermore, shocks in EC and CF demonstrated a positive influence on the INF over the period, whereas the shocks in other variables highlighted a negative influence on INF over the period.

## 6. Conclusions 

This paper examines the association between EC, EG, and sustainable development (SD) among other economic factors for the UFM countries by using the system-GMM model, VECM, VAR model, VDA, and IRA for the period of 1995 to 2014. The economic factors include capital formation (CF), FDI, the labor force (LF), inflation (INF), population (POP), international trade (TR), and financial development (FD).

The estimated results by GMM confirmed the bidirectional causality between EG and EC, no causality between EC and SD, and bidirectional causality between EG and SD. The empirical results of the co-integration test confirmed the co-integration between variables. The VECM results confirmed the long-run equilibrium association in the equations of energy consumption (EC), sustainable development (SD), the labor force (LF), FDI, and inflation (INF). Moreover, the results validated the short-run dynamic association from sustainable development (SD) to EC, EG to sustainable development (SD), EC to CF, energy consumption (EC) to the labor force (LF), sustainable development (SD) to the labor force (LF), and energy consumption (EC) to FDI. Moreover, results also validated the bidirectional short-run causality between EG and the labor force (LF).

Furthermore, the results of VAR model validated the short-run causality from EC to EG, inflation (INF) to EC, EC and inflation (INF) to sustainable development (SD), sustainable development (SD), the labor force (LF) and FDI to the capital formation (CF), EC, capital formation (CF) and FDI to the labor force (LF), energy consumption (EC) and inflation (INF) to FDI, and sustainable development (SD) to inflation (INF).

Based on these results, we can conclude that there is a strong association between EC and EG, and economic growth (EG) is also strongly connected with sustainable development (SD) of the UFM countries. It implies that the higher level of EC can stimulate the growth of the economy, which could help to attain sustainable development (SD) in this region. 

### Policy Implications

The key policy implication of this conclusion is that the energy policies should give full attention not only a causal association between EC and EG but also whether it is temporal or permanent. Consequently, policymakers should formulate policy actions. Policymakers should also consider the possible effects of energy consumption on health, society, and the environment. They should take into consideration the current status of economic and energy sustainability. Consequently, they should highlight deficiencies, and formulate ways to improve the situation. Thus, policymakers need to know about the significances of energy, environmental and economic plans, and their possible effects on the shaping of development and the viability of converting this development into sustainable development (SD).

## Figures and Tables

**Table 1:** Description of Variables.

Variables	Definitions	Measures	Data Source	Reference
EG	Economic Growth	Gross Domestic Product per capita (current US $)	WDI ^1^ database	[55,59]
EC	Energy Consumption	Energy use (kg of oil equivalent per capita)	WDI-database	[54,55]
SD	Sustainable Development	Adjusted savings: net national savings (% of Gross National Income)	WDI-database	[56]
CF	Capital Formation	Gross fixed capital formation (current US $)	WDI-database	[60,61]
LF	Labour Force	Labour force, total (in thousands)	WDI-database	[55,61]
FDI	Foreign Direct Investment	Foreign direct investment, net inflows (current US $)	WDI-database	[55,62]
INF	Inflation	Inflation, consumer prices (annual %)	IMF IFS	[55,60]
POP	Population	Population, total (in thousands)	WDI-database	[55,59]
FD	Financial Development	FD (private sector total credit percentage of GDP)	WDI-database	[55,62]
TR	International Trade	Trade (% of GDP)	WDI-database	[54]

^1^ World Development Indicators (WDI) Database; International Monetary Fund (IMF); International Financial Statistics (IFS).

**Table 2:** Descriptive Statistics.

Variable	Observations	Mean	Standard Deviation	Min.	Max.
LNEG	280	8.5250	0.8423	6.6195	10.0301
LNEC	280	7.4371	0.7014	5.8432	8.4389
LNSD	280	2.6328	0.6689	−2.3026	3.6182
LNFDI	280	22.606	1.4523	16.147	25.2971
LNLF	280	15.345	1.0718	13.3919	17.2158
LNCF	280	23.293	1.2420	19.997	26.3259
LNFD	280	3.4811	0.6981	1.0661	4.6302
LNTR	280	4.4414	0.3995	3.6088	5.2117
LNPOP	280	16.205	1.1683	14.0890	18.3353
LNINF	280	1.8639	1.0000	−2.3026	6.9659

**Table 3:** Panel Unit Root Tests.

Variables	LL	IPS	Fisher-ADF	Fisher-PP
LNEG	−0.62696	3.44169	5.87186	6.18473
LNEC	−2.23769 **	−0.18634	24.6263	28.6548
LNSD	2.54863	−0.20294	26.2688	69.7106 ***
LNFDI	−1.07710	−1.05440	27.8222	50.2990 ***
LNLF	−2.22684 **	2.09847	21.9560	30.9078
LNCF	−3.02963 ***	0.53700	20.2549	13.0840
LNFD	−1.769 **	0.43630	21.0369	22.2443
LNTR	−0.52527	0.38843	18.7796	23.9426
LNPOP	4.12447	3.98439	15.4779	10.6316
LNINF	−0.70326	−0.52159	33.2435	42.5209
∆LNEG	−8.97286 ***	−6.93367 ***	97.3341 ***	96.6577 ***
∆LNEC	−10.5825 ***	−9.70786 ***	140.614 ***	177.995 ***
∆LNSD	−15.4312 ***	−14.1468 ***	200.359 ***	491.487 ***
∆LNFDI	−14.4730 ***	−13.8048 ***	195.412 ***	296.068 ***
∆LNLF	−10.2737 ***	−8.92384 ***	128.190 ***	131.106 ***
∆LNCF	−9.70719 ***	−7.99342 ***	112.422 ***	100.087 ***
∆LNFD	−4.09357 ***	−4.00932 ***	65.1252 ***	65.8534 ***
∆LNTR	−12.6221 ***	−10.2994 ***	143.840 ***	186.585 ***
∆LNPOP	−1.59044 *	−6.11745 ***	97.1069 ***	80.2086 ***
∆LNINF	−12.2562 ***	−11.6901 ***	168.713 ***	253.756 ***

Significance Level: * *p* < 0.1, ** *p* < 0.05, *** *p* < 0.01. LL is Levin-Lin; IPS is Im-Pearson-Shin; ADF is Fisher Augmented-Dickey Fuller; PP is Fisher Philips-Perron.

**Table 4:** Results of System-Generalized Method of Moment (GMM).

Dependent Variables	Model-1	Model-2	Model-3
Independent Variables	EG	EC	SD
	GMM	GMM	GMM
LNEG_L1 ^1^	−0.0378 **	-	-
LNEC_L1	-	−0.0814 ***	-
LNSD_L1	-	-	0.1302 ***
LNEG	-	0.9640 ***	−0.9633 ***
LNEC	0.3111 ***	-	−0.156
LNSD	−0.0822 ***	−0.0462	-
LNCF	0.6897 ***	−0.4585 ***	1.0039 ***
LNLF	−0.6808 ***	2.3185 ***	−1.0339 ***
LNFDI	0.0107	-	−0.0694
LNINF	−0.0055	-	-
LNPOP	-	−1.8152 ***	-
LNFD	-	0.1549 ***	-
LNTR	-	-	−0.0378
Constant	−55.522 ***	101.915 ***	−33.43 *
F	1572.52 ***	417.51 ***	26.91 ***
No. of instruments	112	112	112
AR(1)	−3.73 ***	−3.78 ***	−2.24 **
AR(2)	0.83	0.99	−1.60
Sargan test statistics	237.86 ***	265.18 ***	225.40 ***
Durbin-Wu-Hausman Test	1711.07 ***	46.02 ***	223.21 ***

^1^ L1 is the lag of one period for the said variables; AR(1) is the first order autoregressive process; AR(2) is the second order autoregressive process; Significance Level * *p* < 0.1, ** *p* < 0.05, *** *p* < 0.01.

**Table 5:** Panel Co-integration Test.

Methods		Model-1	Model-2	Model-3
Pedroni				
Within dimension	Panel v-Stat	−2.1372	−1.6250	−0.4579
	Panel rho-Stat	1.7052	2.9038	1.4042
	Panel PP-Stat	−2.0315 **	−5.0732 ***	−9.4390 ***
	Panel ADF-Stat	−1.8712 **	−4.4668 ***	−10.513 ***
Between dimension	Group rho-Stat	3.8023	4.3283	4.0669
	Group PP-Stat	−0.4818	−9.3037 ***	−9.4615 ***
	Group ADF-Stat	−0.3318	−6.1487 ***	−6.7758 ***
Kao	ADF t-Stat	−7.0289 ***	−3.4025 ***	−2.1135 **

Significance Level ** *p* < 0.05, *** *p* < 0.01.

**Table 6:** Panel Vector Error Correction Model (VECM) for the Model (1).

Variables	Model-4	Model-5	Model-6	Model-7	Model-8	Model-9	Model-10
	∆LNEG	∆LNEC	∆LNSD	∆LNCF	∆LNLF	∆LNFDI	∆LNINF
ECT	0.0034	−0.0238 ***	−0.1489 ***	0.0400	−0.0104 *	0.3549 **	−0.8983 *
∆LNEG	-	1.1375	8.7295 **	1.7690	6.7653 **	3.9024	2.3666
∆LNEC	3.4613	-	2.9505	5.5432 ***	4.8125 ***	5.7365 ***	0.1367
∆LNSD	2.2488	5.8068 ***	-	1.4797	9.6283 *	0.9961	0.0809
∆LNCF	2.2354	0.4215	14.627 *	-	2.3476	1.5970	0.9245
∆LNLF	6.4838 **	2.9990	0.2007	2.6936	-	0.0194	0.7085
∆LNFDI	1.2382	0.5274	1.5156	3.8157	0.2713	-	0.5296
∆LNINF	0.9871	1.6072	3.6187	3.9398	7.6775 **	0.6986	-
F-test	2.5822 *	1.4249	2.6858 *	3.5016 *	6.9981 *	3.8220 *	7.2196 *

Significance Level. * *p* < 0.1, ** *p* < 0.05, *** *p* < 0.01.

**Table 7:** Panel Vector Autoregression (PVAR) for the Model (1).

Dependent Variables	Model-11	Model-12	Model-13	Model-14	Model-15	Model-16	Model-17
	LNEG	LNEC	LNSD	LNCF	LNLF	LNFDI	LNINF
LNEG (−1)	0.9218 ***	−0.0122	0.0509	0.0409	−0.0148 **	−0.0393	−0.4053 **
LNEC (−1)	0.0527 **	0.9859 ***	−0.1771 ***	0.0181	−0.0075 **	0.5144 ***	0.1906
LNSD (−1)	0.0137	0.0063	0.6571 ***	0.0355 *	0.0022	−0.0646	−0.1432 **
LNCF (−1)	0.0187	−0.0049	−0.0666	0.8618 ***	0.0165 ***	0.1554	0.2274
LNLF (−1)	−0.0179	0.0030	0.0581	0.1435 **	0.9883 ***	−0.1176	−0.1300
LNFDI (−1)	0.0107	0.0064	0.0360	0.0333 **	0.0022 *	0.6092 ***	−0.0295
LNINF (−1)	−0.0137	−0.0079 *	−0.0451 *	−0.0196	−0.0003	−0.1193 **	0.6813 ***
Constant	−0.0646	0.1371	1.7396 ***	−0.2025	−0.0723 ***	3.9723 ***	0.2634

Significance Level: * *p* < 0.1, ** *p* < 0.05, *** *p* < 0.01.

**Table 8:** Variance Decomposition Analysis for the Model 1 (LNEG).

Period	LNEG	LNEC	LNSD	LNCF	LNLF	LNFDI	LNINF
1	100	0.0000	0.0000	0.0000	0.0000	0.0000	0.0000
2	99.385	0.0347	0.0851	0.0285	0.0055	0.1775	0.2834
3	98.289	0.1087	0.2304	0.0719	0.0154	0.4695	0.8152
4	96.958	0.2151	0.4008	0.1158	0.0275	0.7996	1.4831
5	95.543	0.3486	0.5764	0.1536	0.0405	1.1288	2.2093
6	94.131	0.5047	0.7460	0.1831	0.0535	1.4385	2.9435
7	92.769	0.6801	0.9044	0.2040	0.0660	1.7208	3.6557
8	91.481	0.8724	1.0488	0.2175	0.0777	1.9738	4.3292
9	90.274	1.0796	1.1785	0.2248	0.0885	2.1983	4.9558
10	89.151	1.3001	1.2939	0.2274	0.0984	2.3965	5.5331
